# On Farm Emergency Slaughter and Emergency Killing of Acutely Injured Cattle: Analysis of Guidelines From Five Jurisdictions

**DOI:** 10.3389/fvets.2021.795227

**Published:** 2022-01-24

**Authors:** Paul McDermott, Aideen McKevitt, Alison Hanlon

**Affiliations:** ^1^School of Veterinary Medicine, Veterinary Sciences Centre, University College Dublin, Dublin, Ireland; ^2^Veterinary Department, Department of Environment, Climate Change, and Agriculture, Mayo County Council, Castlebar, Ireland; ^3^School of Agriculture and Food Science, University College Dublin, Dublin, Ireland

**Keywords:** animal welfare, on-farm emergency slaughter, fitness for transport, humane killing, thematic analysis, guidelines, stakeholders, acute bovine injury

## Abstract

**Background:**

In July 2009, the Farm Animal Welfare Advisory Council (FAWAC) in the Republic of Ireland published Animal Welfare Guidelines for the Management of Acutely Injured Animals on Farm in support of a new Irish regulation designed to permit on-farm emergency slaughter (OFES) of cattle. The purpose of this study was to evaluate the FAWAC guidelines, to determine if they remain fit for purpose by comparing them with five guidelines on the management of acutely injured cattle from four jurisdictions purposively selected because of their relevance to OFES, and to represent geographical and organisational diversity; The United Kingdom, Australia, New Zealand and British Columbia/Canada.

**Methodology:**

Content and Thematic Analysis were used to compare the incidence and frequency of themes in the six guidelines using NVIVO 12.

**Results:**

Humane killing and slaughter of animals and the prevention of unnecessary suffering at time of killing were emphasised in all guidelines. Thematic Analysis identified seven primary themes (“parent nodes”): animal welfare; decision tree; certification; legislation; stakeholders; transport and; veterinary ethics. Parent nodes encompassed 26 secondary themes (“child nodes”) including casualty slaughter, on-farm emergency slaughter, euthanasia, unnecessary suffering, animal owner, private veterinary practitioner, official veterinarian and fitness for transport. Guidelines outlined stakeholders' roles in relation to all aspects of managing acutely injured cattle. Results showed similarities between FAWAC, the British Cattle Veterinary Association and the British Columbian/Canadian guidelines in relation to OFES as a method to address acutely injured cattle. OFES is not allowed in Australia or New Zealand as a method of managing acutely injured cattle.

**Conclusions:**

Animal welfare guidelines play a pivotal role in informing all stakeholders involved in the management of acutely injured cattle. Guidelines vary from prescriptive standard operating procedures on actions that should be undertaken for food safety reasons, to descriptive guidance upholding practicalities in relation to equipment and methods to be used in managing acutely injured cattle not meant for human consumption. The FAWAC guidelines remain substantially relevant today and consistent with other welfare guidelines published in the jurisdictions that formed part of the study. However, they need to be reviewed to align with current regulations.

## Introduction

**Figure 1:**
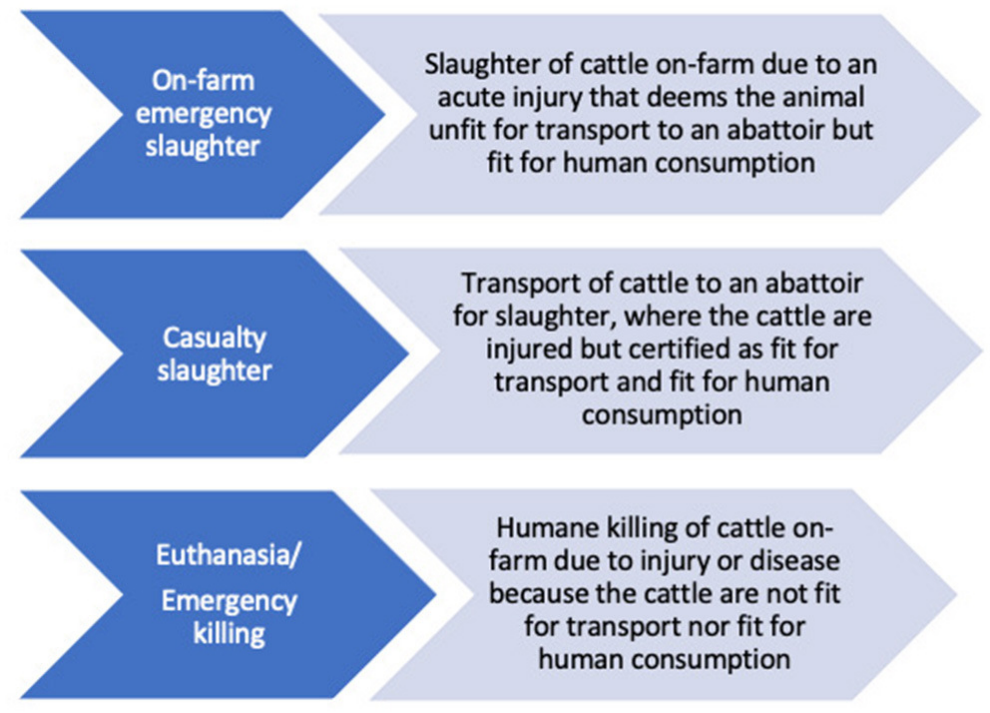
Definitions of procedures for slaughter of injured cattle.

In the Republic of Ireland (hereafter Ireland) there are three potential production outcomes for cattle acutely injured on-farm which are primarily determined by the clinical condition of the animal. These are: on-farm emergency slaughter and casualty slaughter where meat may be used for human consumption, and emergency killing (euthanasia) where the meat from these cattle is not used for human consumption (Figure 1). On-farm emergency slaughter (OFES) is the on-farm slaughter of a healthy animal that has suffered an accident and which, for animal welfare reasons, cannot be transported to an abattoir. Casualty Slaughter (CS) is the slaughter of injured cattle that have been deemed fit for transport to the abattoir under Veterinary Certification. Emergency killing involves the slaughter of cattle where meat is not permitted to enter the food chain for human consumption. The management and production outcomes of acutely injured cattle on-farm and certification requirements for transport and slaughter have been identified as an important ethical challenge for veterinary practitioners (1). To mitigate against the transport of unfit cattle, and the financial losses accrued by emergency killing, a statutory instrument permitting the OFES of cattle in Ireland was published in May 2009 (2). This regulation enables meat from OFES cattle to enter the food chain, provided that ante-mortem examination on farm and post-mortem examination at the abattoir satisfy food safety requirements. In July 2009, the Farm Animal Welfare Advisory Council (FAWAC) in the Ireland published Animal Welfare Guidelines for the Management of Acutely Injured Animals on Farm (3).

For OFES to occur, Food Business Operators must allow carcasses from OFES cattle into their abattoirs for processing. Provided that the animal is deemed suitable for OFES, based on an ante-mortem examination by a Veterinary Surgeon, slaughter is performed, typically on farm, by either a Veterinary Surgeon, or a competent slaughter person as required by Regulation (EC) No 1099/2009 (4). All parts of the carcase must be transported to the abattoir where a post-mortem examination is performed by the Official Veterinarian in Ireland. Prior to the introduction of OFES by European Union (EU) Regulation (EC) No 853/2004 (5), the only permitted options for acutely injured cattle, were emergency killing or transport to an abattoir for CS (6).

Between 2014 and 2015, the European Commission (EC) Directorate-General SANTE division Health and Food Audits and Analysis carried out audits of the systems designed to prevent the transport of unfit livestock to slaughter in 12 EU Member States, as required by Council Regulation (EC) No 1/2005 (7). The key findings were that Member States, when reviewing their controls on the transport of animals to slaughter, should ensure that an effective system is implemented to detect, act upon, and follow up on transport of unfit animals. EU Regulations are legal acts that apply automatically and uniformly to all EU countries as soon as they enter into force, without having to be transposed into national law.

In addition to EU Regulations, guidelines such as those published by FAWAC provide direction to stakeholders on best practise approaches for managing acutely injured cattle.

**Figure 2:**
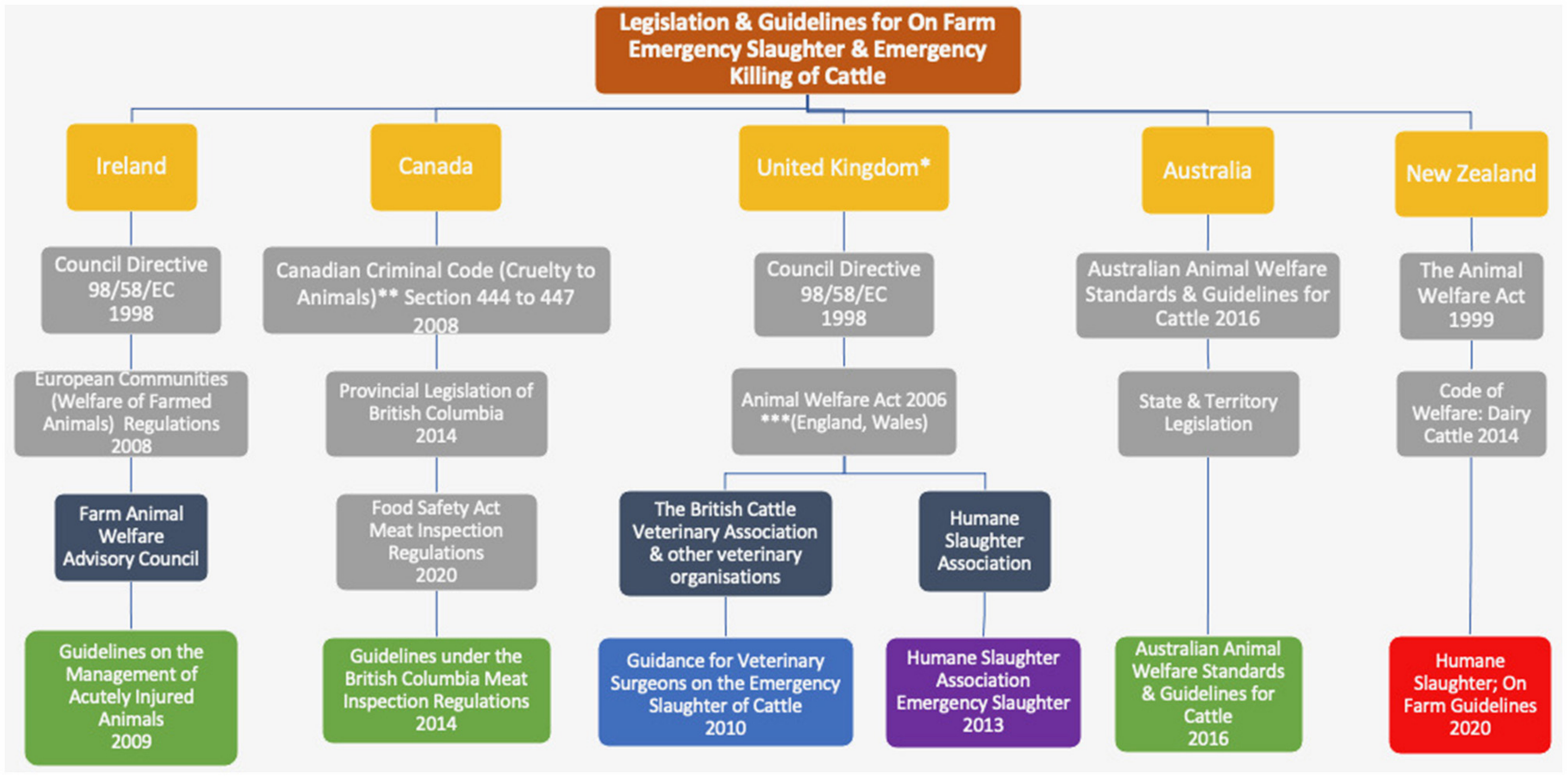
Legislation and guidelines for on farm emergency slaughter and emergency killing of cattle in Ireland, the United Kingdom, Australia, New Zealand and British Columbia, Canada (Yellow) in 2020. Legislation (light grey), organisation (dark grey), competent authority and supporting bodies (green), National Veterinary Organisations (blue), Animal Welfare Charity (purple), industry (red). *The EU regulations were binding in the United Kingdom until 1st january 2021; **Applies federally to the province of Canada; ***Similar legislation pertains in Scotland & Northern Ireland.

The aim of the study was to evaluate and benchmark the FAWAC guidelines on the management of acutely injured cattle to determine whether they were fit for purpose when compared with five other guidelines from four jurisdictions (United Kingdom, British Columbia, Australia, and New Zealand) (Figure 2). Thematic Analysis a qualitative approach used to evaluate the content of documents, and enable a comparison of guidelines, was used to identify and describe concepts in support of animal welfare (8).

## Materials and Methods

### Selection of Study Guidelines

Eleven guidelines about the management of acutely injured farm animals were identified using the One Search facility on the University College Dublin One Connect Portal and Google Scholar. Six search terms were used: animal welfare guidelines, animal welfare legislation, transport of injured animals, on farm emergency slaughter, acute injury of cattle and euthanasia of downer cattle. The data range was 2009 to 2020. The start date corresponded with the introduction of OFES in the Ireland and with the publication of the FAWAC guidelines on the management of acutely injured animals. From the 11 guidelines, 5 were purposively selected because of their relevance to OFES and the management of acutely injured animals, and to represent geographical and organisational diversity and were written in English. Six guidelines were excluded because they did not address the management of acutely injured cattle on-farm but focused on transport (*n* = 4), slaughter at abattoir (*n* = 1) and the care and handling of beef cattle (*n* = 1).

### Content and Thematic Analysis

The six guidelines were downloaded to the software program NVivo 12 (9), for analysis. The content analysis is performed by selecting the content to be analysed.

The guidelines were deconstructed, and their contents coded independently from their original chapter structure. The thematic analysis consisted of assigning a set of nodes (themes), to the guidelines. A theme node is a collection of references from files about a specific theme, topic, concept, idea or experience. The nodes were structured in a hierarchy of parent (primary or overarching theme) and child nodes (secondary, related to the primary or overarching theme). The assignment of nodes was informed by the first author's experience in overseeing the OFES procedure in two abattoirs over a 10-year period.

As a verification step, to validate the coding, the first and third author independently coded the FAWAC guidelines in NVivo, assigning parent and child nodes. The codes from these two separate analyses were then discussed and agreed to create a modified set of nodes. The modified list of nodes was used by the first author to complete the thematic analysis of the remaining five guidelines.

## Results

### Content Analysis

The legal context underpinning the guidelines varied with jurisdiction (Figure 2). The EU regulations were binding in the United Kingdom (UK) until 1st January 2021. Australian Animal Welfare Standards and Guidelines for Cattle were agreed by State and Territory Governments in 2016. The standards are intended to be used as the basis for developing consistent legislation and enforcement across Australia which is the responsibility of jurisdictional (state) governments. They are based on scientific knowledge, recommended industry practise and community expectations and are in the process of being regulated into law by States and Territories (10). In Canada the federal-provincial distribution of legislative powers (also known as the division of powers) defines the scope of the federal and provincial legislatures. These have been identified as exclusive to the federal or provincial jurisdictions or shared by all as per Section 91 of the Constitution Act, 1867. The Animal Care Codes of Practice Regulation (British Columbia Reg 34/2019) applies to all farming operations in British Columbia, Canada, including the production of cattle and cattle by-products (11). In New Zealand, a partnership between the Government and sector groups aimed at improving compliance with the Animal Welfare Act (2013), known as *Safeguarding our Animals, Safeguarding our Reputation*, is a key component of animal welfare strategy (12).

The humane killing/slaughter of animals, emphasising the prevention of unnecessary suffering at the time of killing, was discussed in all guidelines. The role of the competent authorities and the personnel responsible for implementing animal welfare standards varied across the five jurisdictions. In Ireland the Department of Agriculture, Food and the Marine (DAFM) and the Local Authorities (*n* = 31) are the Competent Authorities who implement the OFES protocols (FAWAC) (3). Both have operational procedures to which Official Veterinarians must adhere to regarding OFES. The British Cattle Veterinary Association (BCVA) Guidance for Veterinary Surgeons on the Emergency Slaughter of Cattle (13), states that in the UK, the Department for Environment, Food and Rural Affairs, and the Food Standards Agency, is the competent authority in relation to legislation on animal welfare and information on food hygiene (including Transmissible Spongiform Encephalopathies), respectively. The Food Standards Agency remit covers three countries, England, Scotland and Wales but it has different policy responsibilities within these countries. Food Standards Scotland has responsibility for food policy in Scotland. Devolution in the UK which occurred in 1998 resulted in different policy requirements, accountabilities and priorities across the four countries (14). The Animal Welfare Act 2006 provides legal protection for the welfare of vertebrate animals in England and Wales. Certain provisions of the Act extend to Scotland (Sections 46–50). Similar legislation covers Scotland and Northern Ireland, namely the Animal Health and Welfare (Scotland) Act 2006 and the Welfare of Animals Act (Northern Ireland) 2011 and the Animal Health and Welfare Act 2013 (15).

In British Columbia, The Emergency Slaughter under the Meat Inspection Regulation (BCW) (16), states that the British Columbian Meat Inspection Program outlines the protocols in relation to OFES and these are posted on the Ministry of Agriculture website.

Four guidelines, the BCVA, the Humane Slaughter Association's Guidelines on Emergency Slaughter (HSA) (17), the Australian Animal Welfare Standards and Guidelines for Cattle (AUSW) (18) and the Humane Slaughter, On Farm Guidelines, Dairy New Zealand (NZW) (19) primarily focus on the humane killing of animals and the methods to be used. AUSW and NZW do not address the OFES of cattle for the production of meat that is fit for human consumption as this procedure does not occur in these jurisdictions. In contrast, AUSW provides a broader spectrum covering general animal husbandry as well as slaughter. Both HSA and NZW have a similar structure to AUSW but are shorter and more focussed on emergency killing. Different guidelines use various terms for the killing of cattle: euthanasia, humane killing, emergency killing and emergency slaughter. The FAWAC and BCVA provided decision trees and template certificates for OFES of cattle, for use by Veterinary Surgeons and cattle owners.

### Thematic Analysis

As shown in Table 1 seven parent themes were identified by the authors from the Thematic Analysis: animal welfare, decision tree, certification, legislation, stakeholders, transport and veterinary ethics. Twenty-six secondary themes (“child nodes”) were also identified (Table 1). These included animal welfare framework, casualty slaughter, on-farm emergency slaughter, euthanasia, unnecessary suffering, slaughter process and treatment. The FAWAC guidelines were compared with each of the other guidelines and main similarities and disparities identified. Results for each of the seven parent themes, along with their associated child nodes are discussed separately below.

**Table 1:** A comparison of the parent and child nodes from the thematic analysis of six guidelines for On Farm Emergency Slaughter (OFES) and Emergency Killing of Cattle in Ireland, the United Kingdom, Australia, New Zealand and British Columbia, Canada.

**Parent Nodes**	**Child Nodes**	**Guidelines**					
		**FAWAC**	**BCVA**	**HSA**	**BCW**	**AUSW**	**NZW**
		**Ireland**	**UK**	**UK**	**British Columbia**	**Australia**	**New Zealand**
		**2009**	**2010**	**2013**	**2014**	**2016**	**2020**
Animal welfare	Animal welfare framework	✓				✓	
	Unnecessary suffering	✓	✓	✓	✓	✓	✓
	Slaughter process	✓	✓	✓		✓	✓
	Competent slaughterman	✓	✓	✓		✓	✓
Certification	Ante-mortem examination	✓	✓	✓			
	Food chain information	✓	✓				
	Post-mortem examination	✓	✓		✓		
	Food safety	✓	✓		✓		
	Casualty slaughter	✓	✓	✓			
	OFES	✓	✓	✓	✓		
	Communication	✓	✓		✓		
Legislation		✓	✓	✓	✓	✓	✓
Stakeholders	Cattle	✓	✓	✓	✓	✓	✓
	Animal owner	✓	✓	✓	✓	✓	✓
	Abattoir operator		✓		✓		
	Veterinary surgeon	✓	✓	✓	✓	✓	✓
	Official veterinarian	✓	✓		✓		
	Competent authority	✓	✓		✓		
	Police	✓					
Transport	Suitable vehicle	✓	✓		✓		
	Time	✓	✓		✓		
	Distance	✓	✓				
	Transport/fitness for transport	✓	✓	✓	✓	✓	✓
Veterinary ethics		✓	✓				
Decision tree	Treatment	✓	✓	✓		✓	✓
	Humane slaughter/killing/euthanasia	✓	✓	✓	✓	✓	✓
	Casualty slaughter	✓	✓	✓	✓		
	OFES	✓	✓	✓	✓		
	Treatment	✓	✓	✓		✓	✓

### Parent Themes

#### Animal Welfare

Four child nodes were assigned to the parent theme of animal welfare: animal welfare framework, unnecessary suffering, slaughter process and competent slaughter person. The FAWAC guidelines are structured using the Five Freedoms and was the only guideline that contained an animal welfare framework. Preventing unnecessary suffering and alleviating pain, in the contexts of slaughter, killing, transport and general husbandry are referred to in all of the guidelines (FAWAC, BCVA, HSA, BCW, AUSW, NZW). All guidelines reference that emergency slaughter/ emergency killing was an option to alleviate the animal's suffering in the event of injury or disease and minimise the risks to animal welfare. Four guidelines stated that OFES can be used as a slaughter process on acutely injured cattle (FAWAC, BCVA, HSA, BCW), where the injury precludes transport to the abattoir on regulatory grounds in relation to animal welfare (FAWAC, BCVA and BCW).

All guidelines except BCW outlined the methods that can be used for humane killing/slaughter of cattle. The methods given in the guidelines varied from stunning the animal with a captive bolt followed by exsanguination, use of a close-range firearm or lethal injection by a Veterinary Surgeon (FAWAC, BCVA, HSA, AUSW, NZW). Pithing is referenced in two guidelines stating it may be used to ensure rapid death as an alternative to exsanguination (HSA, NZW). Commission Decision 2000/418/EC (1) banned the use of pithing rods, therefore the use of this technique cannot be used for the stunning and killing of animals within the EU (20).

In addition, the NZW requires a clear written policy on how to slaughter animals. AUSW use the term humane killing for cattle that have to be euthanised and the meat is not used for human consumption while FAWAC and NZW use both humane killing and humane slaughter. The personnel permitted to perform humane slaughter were characterised as a competent, suitably trained slaughterman or a Veterinary Surgeon (FAWAC, BCVA, HSA, AUSW, NZW).

#### Certification

Certification is referenced in four guidelines (FAWAC, BCVA, HSA, BCW). Seven child nodes were assigned to certification (Table 1): Ante-mortem examination, Food Chain Information, food safety, post-mortem examination, CS, OFES and communication. Only two guidelines (FAWAC and BCVA) referred to all seven child nodes, whereas AUSW and NZW did not cover these aspects of certification. The principles of Veterinary Certification are described in the BCVA. The Veterinary Surgeon has a pivotal role in food safety and public health, and these must be a priority when advising farmers (FAWAC, BCVA).

In Ireland and the UK, both OFES and CS require an owner declaration on Food Chain Information providing a record of all veterinary medicinal products or other treatments administered to the animal, within the last 6 months, including dates of administration and withdrawal periods to accompany the carcass or animal to the abattoir (FAWAC, BCVA). Three guidelines provided guidance on certification on OFES (FAWAC, BCVA, BCW). The FAWAC and BCVA require that certificates must accompany OFES cattle to the abattoir and both guidelines provide templates for certification, which must be completed after a Veterinary Surgeon performs a clinical examination of the animal. In the BCW guidelines a “*Document for an Approved Emergency Slaughter on Farm”* for each slaughtered animal(s) must be completed by the Veterinary Surgeon, and they must ensure that this document accompanies the carcass(es) to the abattoir. The Food Business Operator can only approve the acceptance of OFES carcass(es) if a Meat Hygiene Inspector is scheduled to be on site at the establishment for the acceptance of the carcass(es) (BCW). The transportation of an OFES carcass should not involve a food safety risk (BCW). A post-mortem examination must be carried by the Official Veterinarian (or Meat Hygiene Inspector) at the abattoir following OFES to determine the final disposition of the carcass(es) (FAWAC, BCVA, BCW). In the UK, the destination of the carcass will be determined by food safety considerations and that in some circumstances, where the Official Veterinarian has grounds for concern about public health, additional tests should be carried out (BCVA).

Certification for transport of an injured animal to an abattoir for slaughter is referenced in three guidelines (FAWAC, BCVA, HSA). If the animal owner is not sure if the animal is fit for CS, they must communicate with a Veterinary Surgeon (BCVA), and it is a Veterinary Surgeon who must ultimately decide on the suitability of CS (FAWAC). The FAWAC provides a sample veterinary certificate to accompany an injured animal to the abattoir for CS.

Communication between the Veterinary Surgeon and Official Veterinarian, and the animal owner/producer is referenced in three guidelines in relation to OFES (FAWAC, BCVA, BCW). This communication does not obviate the necessity for the required written certification (FAWAC).

#### Legislation

All guidelines provide advice on the legal requirements in relation to the management of acutely injured animals (Table 1). Both the FAWAC and BCVA outline the legislation, national and European, pertaining to animal welfare and food safety. The Australian standards provide a basis for developing and implementing consistent legislation and enforcement across the country and provide guidance for all people responsible for cattle (AUSW). Legislation regarding carcase disposal varies between jurisdictions and Food Business Operators are responsible for implementation of relevant laws (HSA) including the disposal of Transmissible Spongiform Encephalopathies (BCVA) and use of firearms (HSA). Where legislation requires a higher standard than the guidelines, the higher standard will apply (BCVA, AUSW).

Appropriate disposal of carcasses is referenced in three guidelines (HSA, AUSW, NZW) and described in terms of disease risk to other livestock and people, the contamination of waterways and to minimise risks to animal welfare.

#### Stakeholders

Stakeholders in the livestock industry are in a position to make critical choices that directly impact on animal welfare during slaughter and transport. They include farmers, veterinarians, Food Business Operators and those working directly with animals (21). Stakeholders are referenced in all guidelines and seven child nodes were assigned to this theme: cattle, animal owner, abattoir operator, Veterinary Surgeon, Official Veterinarian, competent authority and police. The FAWAC, BCVA, HSA and BCW and NZW reference one or more of the child nodes (Table 1). The guidelines prescribe one or more of the following persons that may be involved in dealing with the emergency killing/slaughter on farm, namely livestock producers, animal-by-products service operators, animal welfare inspectors, animal health officers, agricultural students. Stakeholders' responsibilities are also contained in the guidelines, in terms of animal welfare, OFES, CS, food chain information, euthanasia and transportation. The guidelines are aimed at assisting farmers in arriving at an informed decision on the method of dealing with acutely injured cattle, while having regard to animal welfare and public health (FAWAC, BCVA, HSA, BCW). The farm owner or manager should ensure that designated staff are willing and physically able to carry out humane slaughter (NZW). The AUSW additionally describes the responsibilities of the person in charge that they must take reasonable actions to ensure the welfare of cattle from threats, including extremes of weather, drought, fires, floods, disease, injury and predation.

Two guidelines stated that persons/staff in charge of cattle must have the relevant knowledge, experience and skills to be able to humanely kill cattle or must be under the direct supervision of a person who has the relevant knowledge, experience and skills (AUSW, NZW). In certain jurisdictions only licensed personnel are permitted to perform the OFES (FAWAC, BCVA), including Animal By-Products Collection Services *(“Knackeries”*). The HSA requires the latter to hold certain certification and licences to carry out such on-farm slaughter.

Farmers are advised to call a Veterinary Surgeon when an animal sustains a serious injury or disease (HSA). The Veterinary Surgeon must decide on the most appropriate action when presented with an injured animal and one option is emergency killing and disposal (FAWAC, BCVA). A Veterinary Surgeon will be able to decide if emergency killing is necessary and, if so, kill the animal using a permitted method (HSA). When an emergency slaughter for human consumption is being considered, the farmer must contact the abattoir operator, and the abattoir operator must ensure that the hide of the slaughtered animal is clean (BCVA). The Clean Livestock Policy aims to ensure a consistent approach to categorisation of animals presented for slaughter and to minimise the risk of food poisoning caused by bacteria on dirty coats and fleeces of cattle and sheep (22). The farmer or agent must obtain a Specified Risk Material transportation permit prior to transporting the carcass(es) to the slaughter establishment, as per federal regulation (BCW), and legislation in relation to Transmissible Spongiform Encephalopathies (FAWAC, BCVA). The Official Veterinarian at the designated abattoir must be advised in advance of the arrival of an injured animal and should examine the injured animal upon arrival at the abattoir and perform a post-mortem examination (FAWAC, BCVA, BCW). Temporary Veterinary Inspectors also have responsibility for the post-mortem examination in Ireland (FAWAC).

Appropriate veterinary advice on clinical diagnosis, disease prevention or treatment of cattle should be sought as required (AUSW). A Veterinary Surgeon should be used “*for backup”* in relation to emergency killing if the animal owner is unable to carry out the procedure (NZW).

In some countries, “*knackeries”* are permitted to kill an animal in an emergency and dispose of the carcase (HSA).

#### Transport

Within the EU, regulations state that, “*A person shall not transport an animal by sea, air, road or rail, or cause or permit an animal to be transported, in a way as is likely to cause injury or unnecessary suffering to the animal”* (23). Five guidelines describe general animal welfare provisions during transportation (FAWAC, BCVA, HSA, AUSW, NZW) and four child nodes were identified: suitable vehicle, time, distance, fitness for transport (Table 1). A Veterinary Surgeon must certify that transport to an abattoir for CS is not likely to cause the animal further injury or unnecessary suffering (FAWAC, BCVA, HSA). The AUSW guidelines specify the need to operate in conjunction with Commonwealth, State and Territory legislation, including the Australian Animal Welfare Standards and Guidelines—Land Transport of Livestock, Australian Standards for the Export of Livestock (AUSW). The NZW guidelines additionally refer to the humane killing of an animal that becomes injured during transport, in order to prevent the animal suffering further pain or distress (NZW). Three guidelines reference fitness for transport (FAWAC, BCVA, HSA). The FAWAC states that injured cattle certified fit for transport should be transported without undue delay in an appropriate vehicle, which is not likely to inflict further injury or suffering (FAWAC). The type of vehicle, the provision of, bedding, penning arrangements, and the distance travelled to the destination may have some bearing on the final decision on whether to transport an animal (BCVA). In relation to journey distance when considering CS, the abattoir must be within a reasonable distance (100 Km) (FAWAC). If the animal is to be transported to another location it needs to be fit to travel, a Veterinary Surgeon can help decide about this (HSA). In the UK guidelines, an animal may be transported for treatment if slightly injured (BCVA, HSA).

Three guidelines reference transport of OFES carcases to abattoirs (FAWAC, BCVA, BCW). In the case of OFES, if time delays exceeding 2 h are expected between slaughter and arrival at the abattoir, the carcase must be carried in a refrigerated container (FAWAC, BCVA, BCW). Shorter journey times may be specified depending on the method of transportation (no refrigeration), and weather conditions. A shipment during warm weather may necessitate shipping times as short as 30–45 min (BCW). Carcases must be transported to the abattoir hygienically and without undue delay (BCVA, BCW).

#### Veterinary Ethics

Veterinary ethics is the application of ethical theories, principles, and rules by professionals and paraprofessionals in resolving ethical dilemmas in the practise of veterinary care (24) and adhering to professional ethics is referred to in two guidelines (FAWAC and BCVA) (Table 1). The FAWAC refers to the conflicts of interest of veterinary roles during the OFES process that the Veterinary Surgeon who provides certification for OFES must, on no account, act as post-mortem examination inspector of that animal at the abattoir. The Veterinary Surgeon signing the declaration must ensure that the principles of veterinary certification are fulfilled (BCVA).

#### Decision Tree

A decision tree analysis is a tool supporting a non-parametric approach for problem-solving. It facilitates the evaluation and comparison of the various options and their results (25). Four child nodes were identified for this parent node: treatment, humane slaughter, CS and OFES. Two guidelines provided a decision tree to help the Veterinary Surgeon decide on the course of action when presented with acutely injured cattle (FAWAC, BCVA) (Table 1). The three outcomes are OFES and CS where meat may be passed fit for human consumption and emergency killing which requires disposal of the carcase as animal-by-products (FAWAC, BCVA). Treatment was referenced as a consideration in all guidelines except BCW.

The decision tree in the FAWAC guidelines detail actions based on acceptance or refusal of a Veterinary Surgeon to certify the OFES of cattle and the Official Veterinarian to accept the OFES carcass at a nearby abattoir, where refusal deems euthanasia to be the required outcome. In cases of non-acceptance of euthanasia by the owner, the Official Veterinarian at the abattoir should be informed (FAWAC). Euthanasia is the indicated outcome if a Meat Hygiene Inspector is not present to approve the slaughter (BCW).

Whilst the other guidelines do not provide decision-trees, they advise on decision-making. For example, in Australia the “*person in charge”* of the animal must ensure appropriate treatment for sick, injured, or diseased cattle at the first reasonable opportunity and this includes humane killing (AUSW). Once the decision has been made to discontinue treatment, slaughter must be carried out as soon as possible (NZW).

Four guidelines stated that the choice of slaughter process and the method for killing an animal in an emergency depends on competence of personnel, availability of equipment, legislation, licencing requirements and the location of the animal (BCVA, HSA, AUS, NZW).

## Discussion

An acute injury is an injury that is severe, causes acute pain, has a sudden onset and is usually associated with a traumatic event. Cattle can suffer acute injuries in a variety of ways e.g., slipping on slatted flooring whilst establishing a social hierarchy or during mounting behaviour in mating. The most common injuries are locomotory and fracture of the tibia is the most prevalent (6, 26). In the EU, fitness to travel is a key determinant of whether an animal can be transported. An animal with an acute locomotory injury that is not weight-bearing on all limbs may be considered “unfit” for transport, because transportation is likely to cause unnecessary pain and suffering. Regarding chronic disease, if certified by a Veterinary Surgeon as fit for transport, cattle may be transported to the abattoir for slaughter. An example would be cattle suffering from bovine spastic paresis. At the abattoir, cattle with chronic conditions may be rejected as unfit for human consumption at ante-mortem inspection or condemned at post-mortem inspection.

This study used a combination of Content and Thematic Analyses to compare animal welfare provisions from five jurisdictions globally regarding the management of acutely injured cattle. The main purpose of the study was to review the FAWAC guidelines to establish if they remain fit for purpose by comparison with guidelines from four other jurisdictions. Guidelines were published by three categories of stakeholder: FAWAC, BCW and AUSW guidelines are government instigated documents, developed by or in cooperation with the competent authority and supporting bodies; the BCVA was published by a national veterinary organisation and the HSA UK guideline was published by an animal welfare charity. NZW is an industry publication. The stakeholder role did not make a substantial difference to the content in the guidelines.

The Thematic Analysis demonstrated commonality between the six guidelines. Whilst variation exists in terms of attention given to each of seven primary themes, this may be indicative of the regulations governing the management of acutely injured animals in different jurisdictions. In Australia and New Zealand animal owners take a more proactive part in dealing with injured animals than animal owners in Ireland and the UK, where veterinary advice is generally sought (27). This operational difference may in a large part be due to the logistical challenges associated with geographical range in some regions of Australia and New Zealand.

The analysis also revealed that FAWAC, BCVA and BCW are similar, specifying comparable measures to be followed in relation to certification, operational procedures and practises for OFES. Both UK guidelines (HSA and BCVA) outline in detail the methods and procedures that should be used when dealing with the humane killing of acutely injured animals. These details on how to humanely kill animals are not dealt with by the FAWAC, possibly because there is not a culture of farmers killing their own cattle in Ireland. HSA, AUSW and NZW also provide detailed methods on killing injured cattle. AUSW and NZW are broad-spectrum animal welfare guidelines, while the other four guidelines focus on animal welfare at slaughter and include OFES and humane killing.

The FAWAC, BCW, HSA, and NZW are short documents, while BCVA and AUSW are considerably longer and more complex, both in terms of formulation and overall structure. The Content Analysis revealed that the FAWAC, BCVA, and BCW are more prescriptive than the other guidelines and the verb “must” is used more frequently in these documents than the verb “should.” The verbs “should,” and “must” are frequently used within the same section in HSA, AUSW and NZW which could give the impression that these verbs have interchangeable meanings. The difference between using “must” and “should” is more than semantic as both entail an obligation: “must” prescribes a correct behaviour, whereas “should” allows for personal discretion and professional autonomy (28). In AUSW the standards use “must,” and the guidelines use “should,” in NZW statements that use “must” are regularly but not always referenced by legislation and in the HSA statements that use “must” are not referenced by legislation.

The provision of practical tools such as decision trees and template certificates increase the application value of guidelines. The provision of template certificates can help in providing uniformity and accuracy in certification that can help with the decision-making process. No appendices or decision tree diagrams are present in BCW and a Canadian study in 2019 stated “*Veterinarians also noted a lack of clarity and communication about acceptable animal conditions for OFES and felt they had to rely on their judgement about what was acceptable”* (29). In British Columbia 631 animals underwent OFES in 2015 and represented about 3% of all dairy cows culled in the province (26). Further, to enhance the practical value of guidelines a description of equipment and methods pertaining to humane killing of animals, should be included, as well as the penalties that may be imposed on stakeholders that transport unfit animals and thus cause unnecessary pain and suffering.

Professional obligations of veterinarians were largely absent from all guidelines except the FAWAC, which stated that a Veterinary Surgeon who provides certification for OFES must, on no account, act as post-mortem examination inspector of that animal at the abattoir, thus highlighting a potential conflict of interest in veterinary certification for OFES. For example, there is a potential vested interest of the Veterinary Surgeon to certify an acutely injured cattle fit for transport contrary to the diagnosis, in order to satisfy the client farmer's demands. The FAWAC and BCVA state that the Veterinary Surgeon performing the ante-mortem examination must communicate with the Official Veterinarian at the designated abattoir when considering OFES or CS. This communication can greatly contribute to food safety and animal welfare. Furthermore, clarity on roles and responsibilities within guidelines are required, the FAWAC advice regarding “*If the owner refuses to allow euthanasia, contact the plant Veterinary Inspector*” does not state what actions the Official Veterinarian at the abattoir should take to mitigate against unethical actions.

The FAWAC guidelines were published in 2009 and was one of the first regarding the management of acutely injured cattle and the practise of OFES. It is still relevant today but needs to be updated to take account of new EU hygiene legislation, EU Regulation (EU) 2017/625 (30), and other legislative changes. For example, the provisions requiring trained slaughter persons to have certificates of competence in animal welfare at the abattoir (3), need to be revised. Good governance and best practise suggest guidelines should be reviewed on a regular basis. Generally, guidelines should be reassessed for validity every 3 years (31). The AUSW guidelines were developed in consultation with a wide range of stakeholders including State and Territory governments, livestock industry organisations, animal welfare groups and the general public under the auspices of the Animal Welfare Committee. Stakeholder engagement was also part of developing the FAWAC guidelines, although to a lesser extent than AUSW. FAWAC consists of representatives from the farming industry, competent authorities, research, veterinary bodies, academia. In addition, subject matter experts are co-opted to assist with the development of FAWAC guidelines.

The value and impact of guidelines to support animal welfare are unreported. As a form of dissemination and communication with stakeholders they can be considered as an external factor influencing farmers' views of animal welfare (32), and research is needed to evaluate the impact of guidelines in terms of awareness, usability, knowledge transfer and application by farmers, and veterinary professionals.

## Conclusion

Animal welfare guidelines play a pivotal role in informing all stakeholders involved in the management of acutely injured cattle. The guidelines considered in this study vary from prescriptive standard operating procedures dictating what actions must be undertaken for food safety reasons, to descriptive guidelines upholding the practicalities in relation to equipment and methods to be used in managing acutely injured cattle unfit for human consumption. The FAWAC guidelines remains substantively relevant today and is consistent with those in the other four jurisdictions. However, good governance requires that such guidelines be reviewed and if appropriate, updated on a regular basis to account for new scientific evidence, and changes in the legislative and operational procedures. Furthermore, evaluation of the impact of guidelines should be investigated to determine their use and value as a means of knowledge transfer.

## Data Availability Statement

The raw data supporting the conclusions of this article will be made available by the authors, without undue reservation.

## Author Contributions

PM, AH, and AM conceived and designed the study and collaborated in the collection of the data. PM drafted the manuscript. AH supervised the study and scientific work. AH and AM reviewed the manuscript. All authors contributed to the article and approved the submitted version.

## Funding

The study was funded by a grant from the Department of Agriculture, Food and the Marine, Ireland (Grant No. R20575).

## Conflict of Interest

The authors declare that the research was conducted in the absence of any commercial or financial relationships that could be construed as a potential conflict of interest.

## Publisher's Note

All claims expressed in this article are solely those of the authors and do not necessarily represent those of their affiliated organizations, or those of the publisher, the editors and the reviewers. Any product that may be evaluated in this article, or claim that may be made by its manufacturer, is not guaranteed or endorsed by the publisher.

## Abbreviations

AUSW: Australian Animal Welfare Standards and Guidelines for Cattle
BCVA: British Cattle Veterinary Association Guidance for Veterinary Surgeons on the Emergency Slaughter of Cattle
BCW: Emergency Slaughter Under the British Columbia Meat Inspection Regulation
FAWAC: Farm Animal Welfare Advisory Council
HSA: Humane Slaughter Association, Emergency Slaughter
NZW: Dairy New Zealand, Humane Slaughter, On Farm Guidelines
DAFM: Department of Agriculture, Food and the Marine
CS: Casualty Slaughter
OFES: On-farm emergency slaughter
EC: European Commission
EU: European Union
UK: United Kingdom.
